# Accuracy of endocervical cytological tests in diagnosing preinvasive lesions of the cervical canal in patients with type 3 transformation zone: a retrospective observational study

**DOI:** 10.1590/1516-3180.2019.0435.R1.19112019

**Published:** 2020-04-22

**Authors:** Paula Moskovics Jordão, Fábio Bastos Russomano, Gabriella Tostes Gerbauld, Cecília Vianna de Andrade, Clarice Fraga Esteves Maciel Osorio

**Affiliations:** I MD. Postgraduate Student, Physician and Gynecologist, Instituto Nacional de Saúde da Mulher, da Criança e do Adolescente Fernandes Figueira, Fundação Oswaldo Cruz (FIOCRUZ), Rio de Janeiro (RJ), Brazil.; II MD, PhD. Physician, Gynecologist and Professor of Postgraduate Studies, Women’s Health Sector, Instituto Nacional de Saúde da Mulher, da Criança e do Adolescente Fernandes Figueira, Fundação Oswaldo Cruz (FIOCRUZ), Rio de Janeiro (RJ), Brazil.; III MD, Postgraduate Student, Physician and Gynecologist, Instituto Nacional de Saúde da Mulher, da Criança e do Adolescente Fernandes Figueira, Fundação Oswaldo Cruz (FIOCRUZ), Rio de Janeiro (RJ), Brazil.; IV MD, PhD. Physician, Pathologist and Professor of Postgraduate Studies, Diagnostic and Therapeutic Coordination for Pathological Anatomy and Cytopathology, Instituto Nacional de Saúde da Mulher, da Criança e do Adolescente Fernandes Figueira, Fundação Oswaldo Cruz (FIOCRUZ), Rio de Janeiro (RJ), Brazil.; V MD, MSc. Physician and Cytopathologist, Instituto Nacional de Saúde da Mulher, da Criança e do Adolescente Fernandes Figueira, Fundação Oswaldo Cruz (FIOCRUZ), Rio de Janeiro (RJ), Brazil.

**Keywords:** Sensitivity and specificity, Mass screening, Pathology, Colposcopy, Uterine cervical neoplasms, Endocervical lesions, Endocervical brushing, Type 3 transformation zone

## Abstract

**BACKGROUND:**

Cervical cancer screening in Brazil is done using Pap smears. Women who are most likely to have a preinvasive lesion or cervical cancer are immediately referred for colposcopy.

**OBJECTIVE:**

The aim of this study was to evaluate the diagnostic performance of endocervical cytological tests in diagnosing preinvasive cervical lesions in women with initial high-grade squamous intraepithelial lesions (HSIL), or atypical squamous cells in which high-grade lesions could not be ruled out (ASC-H), or atypical glandular cells (AGC), and whose colposcopy did not show any abnormalities, with no fully visible transformation zone (types 2 and 3).

**DESIGN AND SETTING:**

Retrospective observational study conducted in Rio de Janeiro, Brazil.

**METHODS:**

Data from women who came to the cervical pathology outpatient clinic between January 2012 and April 2017 were analyzed. The results from endocervical cytological tests were compared with the final diagnosis, which was obtained through examination of a surgical specimen or, among women who did not undergo an excisional procedure, after cytological and colposcopic follow-up for two years.

**RESULTS:**

We included 78 women. The sensitivity of endocervical cytological tests was 72.7%; specificity 98.5%; positive and negative predictive values 88.9% and 95.6%, respectively; and positive and negative likelihood ratios 48.7 and 0.28.

**CONCLUSION:**

Endocervical cytological tests are simple, inexpensive and noninvasive, and form a reliable method for determining management among patients with HSIL, ASC-H and AGC cytological findings and negative colposcopic findings without visualization of the squamocolumnar junction.

## INTRODUCTION

Cervical cancer is the fourth most common type of cancer among women worldwide, accounting for 570,000 new cases and 305,000 deaths in 2018.^1^ In Brazil, 16,370 new cases were expected for that same year. In 2016, it was the third most frequent tumor, and the fourth largest cause of cancer-related death, excluding non-melanoma skin cancer.^2^ While mortality has declined over recent years,^3^ cervical cancer remains a public health problem, and reducing its incidence and mortality is still an ongoing challenge.

Periodic conventional Pap smears remain the most widely adopted strategy for cervical cancer screening in Brazil. When the results from this test show alterations that are most likely to represent the presence of cancer or its preinvasive lesions, immediate referral for colposcopy is necessary.^4^ These lesions can include presence of atypical squamous cells in which high-grade lesions cannot be ruled out (ASC-H); atypical glandular cells (AGC); high-grade squamous intraepithelial lesions (HSIL); high-grade squamous intraepithelial lesions in which microinvasion cannot be ruled out; invasive squamous carcinoma; adenocarcinoma in situ (AIS); and invasive cancer.

When the squamocolumnar junction (SCJ) is not completely visible (type III transformation zone, TZ), colposcopy cannot rule out the presence of endocervical disease. In these cases, the most accurate method for doing so, or for treating it if present, is type III excision (cervical conization). However, this procedure increases the risk of obstetric complications^5^ or other disorders, such as endocervical canal stenosis, thereby impairing these patients’ follow-up,^6^ and possibly compromising their quality of life.

Alternatively, endocervical cytological tests may aid diagnosis and management, often preventing conization, especially if colposcopy does not show any alteration. In Brazil, this test is recommended for women with the abovementioned cytopathological diagnoses, type III TZ and without colposcopic abnormal findings, but for whom there is a lack of definitive evidence.^4^

A systematic review of the literature using the terms “endocervical canal cytology”, “endocervical canal”, “endocervical canal cytology accuracy”, in PubMed, SciELO (Scientific Electronic Library Online) and EMBASE (Excerpta Medica Database), found only one study evaluating the performance of endocervical cytological tests in a similar situation, albeit using a non-conventional processing method.^7^

A new search for papers in PubMed using the terms “endocervical brushing” and “accuracy” returned nine papers. The most recent of these evaluated cytology tests in a liquid medium or compared sample collection methods. However, none of these studies provided an adequate gold standard for estimating the accuracy of detection of preinvasive endocervical canal disease in the situation studied here, i.e. baseline cytological assessment of ASC-H, HSIL or AGC, with no fully visible transformation zone and no abnormal colposcopic findings.

The lack of evidence regarding endocervical cytological test performance in this situation causes considerable uncertainty regarding its usefulness in clinical practice for defining which women should undergo an excisional procedure and which of them just need to be kept under follow-up.

## OBJECTIVE

The aim of this study was to establish the diagnostic performance of conventional endocervical cytological tests for diagnosing preinvasive and invasive cervical lesions in women with Pap smears showing ASC-H, HSIL or AGC without colposcopic abnormal findings and with the SCJ not fully visible (type III TZ).

## METHODS

This was an observational study, in which information was obtained from our institution’s cervical pathology outpatient database, complemented by data extracted from medical records.

Women who first came to our clinic between January 2012 (after the first publication of the Brazilian guidelines^8^ recommending the use of endocervical cytological tests in these situations) and April 2017 were considered eligible. Data relating to the initial cytological tests, colposcopic evaluation, endocervical cytological test result and the final diagnosis were used.

The baseline cytological data were obtained conventionally at primary healthcare facilities of the Brazilian National Health System (Sistema Único de Saúde, SUS). At these facilities, cytological laboratories are subjected to internal and external quality monitoring, as recommended.^9^

Women with an indication for colposcopy are referred for diagnosis and treatment at units that can provide care for cases of higher complexity, such as the institution where the study was carried out. This institution has one of the referral units for diagnosing and treating preinvasive cervical lesions in the state of Rio de Janeiro.

Endocervical cytological specimens were obtained by means of endocervical brushing, which was done after the colposcopic examination. These specimens were processed using the conventional method (Papanicolaou).

Type III excision procedures (cervical conizations) were performed by means of electrosurgery, using a 2 cm loop electrode or a straight wire electrode to reach a depth of at least 2 cm in the endocervical canal.^10^ The length of follow-up for women who did not undergo the excisional procedure was two years, with semiannual cytological tests and colposcopy. After this period, if there was no evidence of preinvasive disease or cancer, they were discharged for cytological follow-up at the primary healthcare facility. Any new tests with results suggesting preinvasive lesions or cancer would lead to an excisional procedure, as recommended in the current Brazilian guidelines.^4^

The colposcopies, excisional procedures, cytopathological tests and histopathological examinations were performed by the highly experienced colposcopists, cytologists and pathologists at Instituto Nacional de Saúde da Mulher, da Criança e do Adolescente Fernandes Figueira/Fundação Oswaldo Cruz (IFF/FIOCRUZ).

### Study population

All the women who were referred to our institution from primary healthcare facilities and were received presenting either of the following were included in this study: (a) initial HSIL or ASC-H cytological and colposcopic findings without significant abnormalities, and with non-fully visible or partially visible SCJ (type III TZ); or (b) with initial AGC cytological findings, regardless of the type of TZ or abnormal colposcopic findings. In all these cases, the women underwent endocervical cytological tests to define the case management.

Patients whose endocervical tests done during the initial approach at our institution showed the same positive results as in the referral test, or more relevant results than those of the referral test, were considered positive. It was recommended that these patients should undergo an excisional procedure (type III excision, i.e. conization), although in reality not all of these patients underwent the procedure, while some others did so even though the new test had been negative. For patients who were considered negative (i.e. those with other results), it was recommended that they should be followed up for at least two years after the initial assessment.

Pregnant women were not included in this study because of the difficulty in following them up and the impossibility of performing an immediate excisional procedure. Women who were lost to follow-up, despite repeated attempts to contact them via letters or by telephone, were excluded.

### Diagnostic criteria

Endocervical cytological tests that showed diagnoses of HSIL, ASC-H or AGC were considered positive, since these results, according to the Brazilian Guidelines for Cervical Cancer Screening, indicate that an excisional procedure is needed in order to obtain the diagnosis. In cases in which squamous preinvasive disease is present, no further treatment is needed.^4^ There were no cases with AIS in the endocervical cytological findings, or with cancer. All others, in which satisfactory material was obtained (including at least squamous and glandular or metaplastic epithelium) and did not show any of these cytological diagnoses, were considered negative.

### Gold standard

The results from the endocervical cytological tests were compared with the definitive diagnosis that had been obtained through one of the following methods: (a) among women undergoing type III excision (conization), histopathological examination of the specimen obtained as part of the initial approach (through a positive endocervical cytological test); or (b) among women undergoing cytological and colposcopic follow-up (not undergoing excisional procedure as the initial approach), negative results from cytological and colposcopic examinations that were performed semiannually for two years or from histopathological examination of the specimen obtained through an excisional procedure performed after a new cytological or colposcopic examination showed a suspected preinvasive lesion during follow-up.

The final diagnosis took into consideration whether a preinvasive cervical lesion was present: either squamous (cervical intraepithelial neoplasia, CIN 2 or 3) or glandular (AIS). No cases of cancer were found. All other diagnoses were taken to be absence of preinvasive disease or cancer.

### Data analysis

The data on the women included in this study were transferred to a spreadsheet. After checking for nonconformities, they were corrected and supplemented using information from the physical medical charts. Performance measurements were calculated from contingency tables that were constructed using the Statistical Package for the Social Sciences (SPSS), version 21.

### Ethical matters

This was a retrospective study. The stored data were identified only by the numbers of the medical records, thus preserving the women’s identity. This study was approved by the local research ethics committee under the number CAE 03847218.0.0000.5269, on December 20, 2018.

## RESULTS

Ninety-one women seen between January 2012 and April 2017 were found to be eligible. Of these, 13 were excluded because they had not completed a two-year follow-up. Hence, 78 patients remained in the study, among whom nine women were deemed to be positive, since they had endocervical cytological findings that maintained the cytological diagnosis of their referral or another result suggestive of preinvasive disease. These women underwent type III excision.

**Figure 1.**
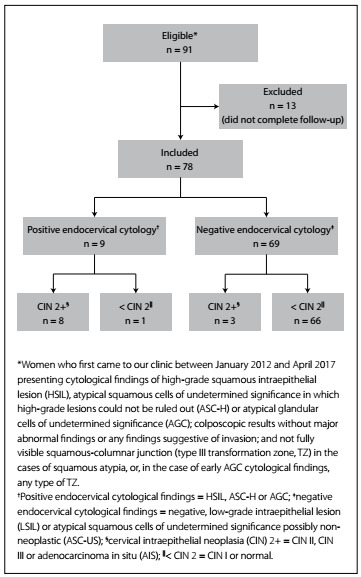
Study flowchart (IFF/FIOCRUZ, Rio de Janeiro, 2012-2017).

The remaining 69 women whose endocervical cytological findings suggested diagnoses of lesser importance than the initial one were deemed to be negative (Figure 1). Twelve women, out of the 78 women who remained in the study, underwent an excisional procedure after the early colposcopy (three despite negative endocervical cytological results) and the other 66 (66/78) underwent semiannual cytological and colposcopic follow-up for two years. During this follow-up, only one patient had new positive cytological findings (AGC), at the six-month evaluation. This woman underwent an excisional procedure that confirmed the presence of preinvasive disease (CIN 3) and her case was considered to be a false negative from the endocervical cytological test that had been performed during the initial assessment. A total of 13 women underwent an excisional procedure (12 after the initial approach and one during follow-up). Among these, 11 had findings compatible with a preinvasive lesion (CIN 2-3 or CIN 2-3 with concomitant AIS) and two were negative (no abnormalities or CIN 1).

The characteristics of the women included, the initial colposcopic findings and the final diagnoses are shown in Table 1. Baseline cytological tests showing AGC were more frequent; the patients’ mean age was close to 50 years; and HSIL was the preinvasive disease most often found among the women with baseline cytological tests.

**Table 1.** Characteristics of the women included, their initial examinations and final diagnoses (IFF/FIOCRUZ, Rio de Janeiro, 2012-2017)

Baseline cytological findings*	AGC n (%)	ASC-H n (%)	HSIL n (%)	Total
Mean age (SD)	35 (44.9)	27 (34.6)	16 (20.5)	78
	45.4 (11.3)	52.1 (11.1)	47.7 (12.3)	48.2 (11.7)
Parity				
Nulliparous (%)	3 (8.6)	0 (0)	0 (0)	3 (7.3)
1-2 children (%)	7 (20.0)	11 (40.7)	1 (6.3)	19 (46.3)
3 or more children (%)	7 (20.0)	7 (25.9)	5 (31.3)	19 (46.3)
Unknown	18 (51.4)	9 (33.3)	10 (62.5)	37 (47.4)
Colposcopic findings				
Major abnormal^†^ (%)	1 (2.9)	NA	NA	1 (1.3)
Minor abnormal (%)	9 (25.7)	8 (29.6)	1 (6.3)	18 (23.1)
Negative^‡^ (%)	25 (71.4)	19 (70.4)	15 (93.7)	59 (75.6)
Final diagnosis^§^				
CIN 2-3	1 (2.9)	3 (11.1)	6 (37.5)	10 (12.8)
CIN 2-3 + AIS	0 (0)	1 (3.7)	0 (0)	1 (1.3)
Negative	34 (97.1)	23 (85.2)	10 (62.5)	67 (85.9)

AGC = atypical glandular cells; ASC-H = atypical squamous cells in which high-grade squamous intraepithelial lesion cannot be ruled out; HSIL = high-grade squamous intraepithelial lesion; SD = standard deviation.

*Result from cytopathological examination that motivated the referral for colposcopy; ^†^applies only to women with baseline cytological finding of AGC, since the inclusion criterion was absence of major findings in other women with cytological findings of ASC-H or HSIL (NA = not applicable); ^‡^includes normal, nonspecific and miscellaneous findings (endocervical polyps); ^§^obtained through excisional procedure or through semiannual cytological and colposcopic follow-up for two years (positive = CIN 2-3 or CIN 2-3 + AIS [CIN 2 or 3 concomitant with adenocarcinoma in situ]; negative = CIN 1 or absence of intraepithelial disease).

Table 2 shows the results from the endocervical cytological tests and the gold standard that was established. It also shows the diagnostic performance measurements for all the women according to the nature of the cytological atypia (squamous or glandular) that were initially seen in these women. Some measurements could not be calculated due to lack of cases.

**Table 2.** Distribution of endocervical cytological results, final diagnoses and diagnostic performance measurements regarding endocervical cytological testing (IFF/FIOCRUZ, Rio de Janeiro, 2012-2017)

Endocervical cytological findings	Preinvasive disease present*	Preinvasive disease absent*	Total	Sensitivity % (95% CI)	Specificity % (95% CI)	PPV % (95% CI)	NPV % (95% CI)	LR+	LR-
All cases				72.7 (46.4-99.0)	98.5 (95.6-100)	88.9 (68.4-100)	95.6 (90.8-100)	48.7	0.28
Positive^†^	8	1	9	-	-	-	-	-	-
Negative^‡^	3	66	69	-	-	-	-	-	-
Total	11	67	78	-	-	-	-	-	-
Squamous abnormality^§^				80.0 (55.2-100)	96.9 (91.1-100)	88.9 (68.4-100)	94.1 (86.2-100)	26.4	0.21
Positive^†^	8	1	9	-	-	-	-	-	-
Negative^‡^	2	32	34	-	-	-	-	-	-
Subtotal	10	33	43	-	-	-	-	-	-
Glandular abnormality^||^				^¶^	100 (^¶^)	^¶^	97.1 (91.6-100)	-	-
Positive^†^	0	0	0	-	-	-	-	-	-
Negative^‡^	1	34	35	-	-	-	-	-	-
Subtotal	1	34	35	-	-	-	-	-	-

PPV = positive predictive value; NPV = negative predictive value; LR+ = positive likelihood ratio; LR- = negative likelihood ratio.

*Compounding of histopathological examinations on surgical specimens in women who underwent an excisional procedure or semiannual cytological and colposcopic follow-up for two years; ^†^Cytological findings of high-grade squamous intraepithelial lesion (HSIL), atypical squamous cells in which high-grade squamous intraepithelial lesion (ASC-H) cannot be ruled out, or atypical glandular cells (AGC); ^‡^negative cytological findings or cytological findings of atypical cells of undetermined significance that are possibly non-neoplastic (ASC-US) or low-grade squamous intraepithelial lesion (LSIL); ^§^baseline cytological and colposcopic findings of HSIL or ASC-H; ^||^baseline cytological and colposcopic findings of AGC; ^¶^not possible to calculate.

## DISCUSSION

This study was conducted using retrospective data that were obtained from the clinical records of women who had been referred from primary care units in municipalities of the state of Rio de Janeiro, for diagnosis and treatment of preinvasive cervical lesions. All of the procedures performed were within our usual care. Our institution is one of the units that receive these women, and its practices follow the recommendations of the Brazilian Guidelines for Cervical Cancer Screening.^4^

The patients’ mean age was 48.2 years. The low number of nulliparous women was as expected, given the inclusion criteria (especially regarding no visible SCJ). Because of the retrospective design of this study, little demographic information was available, but the characteristics of our sample resembled those of the regular users of public healthcare units in Rio de Janeiro.

Both the baseline cytological tests and the endocervical cytological tests done during colposcopy were performed by means of the conventional method that is predominantly used in laboratories that provide services to SUS. Accordingly, the results observed here apply to most Brazilian referral services that follow the same recommendations.

In our sample and our setting, endocervical cytological tests among women with type III TZ without abnormal colposcopic findings and with HSIL, ASC-H, and AGC cytological screening findings was most useful when this diagnosis was negative or when the results were less likely to represent a preinvasive lesion (ASC-US or LSIL). In these situations, the test ruled out disease in almost all healthy women, i.e. it diagnosed almost all true negatives. This can be inferred from the specificity of 98.5% and the negative predictive value (NPV) of 95.6% (Table 2).

The observed sensitivity of 72.7% also shows that endocervical cytological tests are also useful when positive, i.e. they show a high probability of being positive among women who truly have preinvasive disease. Although these tests do not detect all cases, they adequately indicate the excisional procedure most of the time, as expressed by the positive predictive value (PPV) of 88.9% (Table 2). It can also be considered that the excisional procedure was still useful when it did not show the presence of preinvasive lesions or cancer, since it served as a diagnostic method, thereby ensuring the absence of endocervical preinvasive disease in women who had had at least two positive cytological examinations (baseline and the one done during early colposcopy).

The large confidence intervals that were observed in the diagnostic performance measurements (Table 2) resulted from the small sample size. Despite the long study period of five years, few women met the requirements for using endocervical cytological findings as a predictor for endocervical disease.

The exclusion of women who had not completed two years of follow-up after negative endocervical cytology results contributed to this limitation. However, this also had the aim of preventing the bias that would result from their inclusion, since they would not have had enough time to show one of the outcomes.

We calculated the diagnostic performance measurements separately between women whose baseline cytological tests showed squamous atypia (ASC-H or HSIL) and those whose tests showed results of glandular nature (AGC), in order to differentiate diagnostic performance according to the nature of the atypia seen in the baseline cytological tests. The performance of the endocervical cytological tests among the women whose baseline cytological findings consisted of squamous atypia was like that obtained overall, among all the women included in this study. Regarding the situations in which the baseline cytological tests showed glandular atypia, it was only possible to calculate the specificity and the NPV, since there were no cases of positive endocervical cytological data collected during early colposcopy in this group. The measurements calculated also pointed towards excellent specificity and high NPV values. However, all of these measurements were less accurate because they were calculated among only part of the total sample.

Since the predictive values may vary with the prevalence of the disease in a specific population, these results can only be applied to services with the same patient profile as ours, i.e. those that only receive patients who are referred and approached in accordance with the current guidelines, from populations with the same disease prevalence, which is very difficult to ensure. Thus, we chose to calculate the positive likelihood ratio (LR+) and negative likelihood ratio (LR-), which are measurements that provide predictions of the presence or absence of disease, regardless of disease prevalence. In our study when the test was positive, LR+ was 48.7; and when negative (LR-), 0.28. These measurements can be considered to represent excellent performance, since they give rise to a significant change in the pretest probability of the presence of a preinvasive or invasive disease when the result from the endocervical cytological test is positive and exceeds the threshold for making the decision to undertake an excisional procedure. Conversely, a negative result provides a safe possibility for follow-up. This safety was emphasized by the absence of invasive carcinoma in our sample and the possibility of detection of preinvasive disease during the follow-up, as occurred in one case in our study.

Negative predictors are essential because they reduce the risk of overtreatment, which could lead to reproductive problems in the future for these women (such as miscarriage and premature birth),^5^ along with cervical canal stenosis. Such situations would impair the follow-up among these women or lead to secondary dysmenorrhea or even hematometra, thus requiring new surgical procedures.^6^

The mean age that was observed in our sample resulted from the inclusion criteria through which the women were selected. This characteristic did not represent bias, since these were the women who would benefit most from endocervical cytological testing, to whom these results can be applied, following the recommendations of the current Brazilian guidelines.^4^

The low frequency of preinvasive disease among women with baseline cytological findings of AGC (1/35; 2.8%), compared with the frequency observed among women with baseline cytological findings of ASC-H or HSIL (10/43; 23.2%), may have been related to the higher frequency of preinvasive squamous cervical lesions in general and the greater difficulty of making a cytological diagnosis of AGC.

Our study showed results that were close to those obtained by the only paper identified in the systematic review cited above. In the study thus identified, Goksedef et al.^11^ compared the performance of endocervical brushing with that of cervical curettage. In their study, endocervical cytological tests showed slightly higher sensitivity than what we observed (83.3%), and similar specificity (96.5%). The difference in sensitivity may be explained in terms of random error due to the small sample size of both studies, in association with other variations, such as their inclusion criteria, since they only included women with LSIL as referral cytological results, among whom preinvasive lesions are known to present low prevalence. Moreover, those authors used a different brushing technique for sample collection: the material obtained through brushing was fixed in formaldehyde and processed for embedding in paraffin blocks, followed by histological analysis (rather than using conventional cytological analysis). This may produce performance differing from that of the usual technique. Hence, the results reported by Goksedef et al.^11^ can only be extrapolated to sample collections using the same method. Apart from these factors, they did not define the method and depth of treatment excision that would be considered the gold standard in the study (which may have missed some positive results in the case of endocervical lesions) and did not find the diagnoses that were eventually obtained during the follow-up, when results that had been lost in the early evaluation could be obtained, as we did in our study, which decreased the sensitivity of the test in our research.

## CONCLUSION

We can conclude that, in our practice, conventional endocervical brushing is a reliable test for determining the management that should be used among patients with HSIL, ASC-H and AGC screening cytological and colposcopic tests without abnormal findings and without a full view of SCJ (Type III TZ) as recommended in the Brazilian Guidelines for Cervical Cancer Screening.

The likelihood ratios found ensured that endocervical cytological testing presented good diagnostic performance, regardless of the prevalence of the disease among these women.

Further studies with larger sample sizes and in other scenarios are required to increase the accuracy and consistency of these results and to strengthen the recommendations for using this test to investigate women in the situation studied here.

Endocervical cytological testing is a simple low-cost noninvasive method that should be encouraged in referral centers that receive these patients. Its use can avoid unnecessary hospitalizations and invasive procedures, thereby reducing the potential complications of type III excision (conization).
